# Lymphangiogenesis in Regional Lymph Nodes Is an Independent Prognostic Marker in Rectal Cancer Patients after Neoadjuvant Treatment

**DOI:** 10.1371/journal.pone.0027402

**Published:** 2011-11-07

**Authors:** Christiane Jakob, Daniela E. Aust, Birgit Liebscher, Gustavo B. Baretton, Kaustubh Datta, Michael H. Muders

**Affiliations:** 1 Institute of Pathology, University Hospital Carl Gustav Carus, University of Technology, Dresden, Germany; 2 Department of Biochemistry, University of Nebraska Medical Center, Omaha, Nebraska, United States of America; Children's Hospital Boston & Harvard Medical School, United States of America

## Abstract

One of the major prognostic factors in rectal cancer is lymph node metastasis. The formation of lymph node metastases is dependent on the existence of a premetastatic niche. An important factor preceding metastasis are lymph vessels which are located in the lymph node. Accordingly, the occurrence of intranodal lymphangiogenesis is thought to indicate distant metastasis and worse prognosis. To evaluate the significance of lymph node lymphangiogenesis, we studied formalin fixed, paraffin embedded adenocarcinomas and regional lymph nodes of 203 rectal cancer patients who were treated with neoadjuvant radiochemotherapy and consecutive curative surgery with cancer free surgical margins (R0). Regional lymph node lymph vessels were detected by immunohistochemistry for podoplanin (D2-40). Our results show that the presence of lymphatic vessels in regional lymph nodes significantly affects the disease-free survival in univariate and multivariate analyses. In contrast, there was no correlation between peritumoral or intratumoral lymph vessel density and prognosis. Indeed, our study demonstrates the importance of lymphangiogenesis in regional lymph nodes after neoadjuvant radiochemotherapy and consecutive surgery as an independent prognostic marker. Staining for intranodal lymphangiogenesis and methods of intravital imaging of lymphangiogenesis and lymphatic flow may be a useful strategy to predict long-term outcome in rectal cancer patients. Furthermore, addition of VEGF-blocking agents to standardized neoadjuvant treatment schemes might be indicated in advanced rectal cancer.

## Introduction

Preoperative (neoadjuvant) radiochemotherapy followed by curative surgery has become standard treatment in locally advanced rectal cancer [1]. The histopathological tumor regression as a correlate for local treatment efficacy varies from no to minimal regressive changes to complete response [2], [3]. The lymph node status after neoadjuvant radiochemotherapy, however, remains the most important independent prognostic factor for disease-free survival, as patients with post-therapeutic positive lymph nodes (ypN+) have an unfavourable prognosis irrespective of regression of the primary tumor [2], [3], [4], [5], [6]. Thus, inhibition of regional lymph node metastases might be an effective future avenue in treating rectal cancer patients. Therefore, efforts have been made to uncover the mechanisms of lymph node metastasis. It has been suggested that the induction of lymphangiogenesis in the primary tumor and in lymph nodes significantly contributes to tumor metastases [7], [8]. In this study we have validated lymphangiogenesis in regional lymph nodes as a predictor for poor prognosis and facilitator for distant metastasis in a cohort of neoadjuvant treated advanced rectal cancer patients. It is a better predictor than peri- and intratumoral lymph vessel densities or standardized tumor regression grading systems for this group of rectal cancer patients.

## Results

### Clinicopathological characterization of the evaluated cohort

All patients underwent rectum resection with total mesorectal excision after neoadjuvant radiochemotherapy. In all patients a locally curative resection was achieved (R0-situation). Pathological staging is reported in Table 1. Complete tumor regression [9] was observed in 10 cases (5%). 57 patients experienced tumor regression grade 3 (28%). 85 patients showed tumor regression grade 2 (42%) and 48 patients had tumor regression grade 1 (23%). No tumor regression was observed in 3 patients (1.5%). At median follow-up of 223 weeks (95% confidence interval 216.9 to 247.3 weeks) 38 patients had disease recurrence, 32 developed distant metastasis and 6 local recurrences. Distant metastases were detected in the following organs: hepatic 15 (HEP), pulmonary 6 (PUL), distant non-regional lymph nodes 3 (LYM), pleura 1 (PLE), skin 1 (SKIN), peritoneum 1 (PER), bone 2 (OSS), hepatic and pulmonary 2 (HEP and PUL) and others 1 (OTH). In these cases disease recurrence and distant metastases were confirmed by pathologic evaluation and examination. In eight cases clinical information (accessed by a documentation assistant) was used to detect disease recurrence.

**Table 1:** Characterization of the evaluated cohort and the correlation of the presence of lymph node lymph vessels (nLV+) with clinicopathological parameters.

Variable	Numbers	Detectable LVD in lymph nodes (nLV+)	p-value
Sex			0.4052 (Pearson)
Male	148 (73%)	71 (47%)	
Female	55 (27%)	30 (55%)	
Age, years			0.7188 (Pearson)
≤70	112 (55%)	57 (51%)	
>70	91 (45%)	44 (48%)	
yUICC stage			***0.0026 (Pearson)***
0	10 (5%)	3 (30%)	
I	66 (33%)	22 (33%)	
IIA	58 (29%)	31 (53%)	
IIB	3 (1%)	1 (33%)	
IIC	1	1 (100%)	
IIIA	9 (4%)	8 (89%)	
IIIB	32 (16%)	17 (53%)	
IIIC	5 (2%)	5 (100%)	
IVA	19 (9%)	13 (68%)	
Relapse			***0.0171 (Pearson)***
yes	46 (23%)	30 (65%)	
No	157 (77%)	71 (45%)	

**Figure 1:**
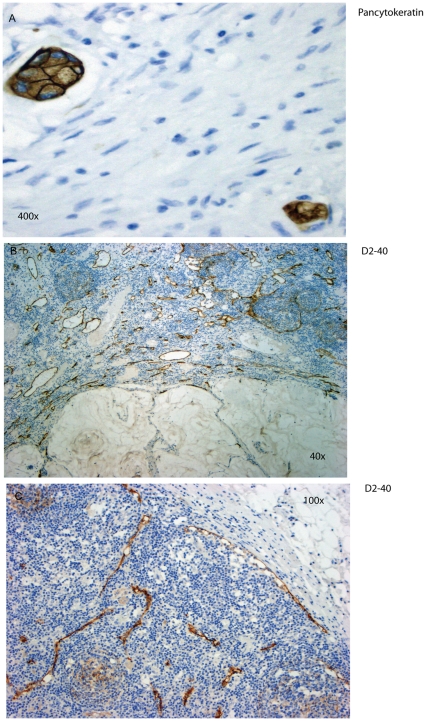
Immunohistochemistry of regional lymph nodes. A. Immunohistochemistry for Pancytokeratin to detect lymph node micrometastases. B. Podoplanin staining with D2-40 in a lymph node with metastasis. The red to brown staining marks lymph vessels. C. Podoplanin staining with D2-40 in a lymph node without metastasis.

To detect metastatic tumor cells in lymph nodes we used standard HE staining as well as Pan-Cytokeratin staining using a commercially available monoclonal antibody (Dako Inc) in at least three serial sections of the lymph node. A median of 14 regional lymph nodes per case was studied. The presence of Pancytokeratin positive tumor cells indicated lymph node metastasis (Fig. 1A).

### Association of regression of primary tumor and lymph node metastasis with clinicopathological parameters and prognostic parameters

**Figure 2:**
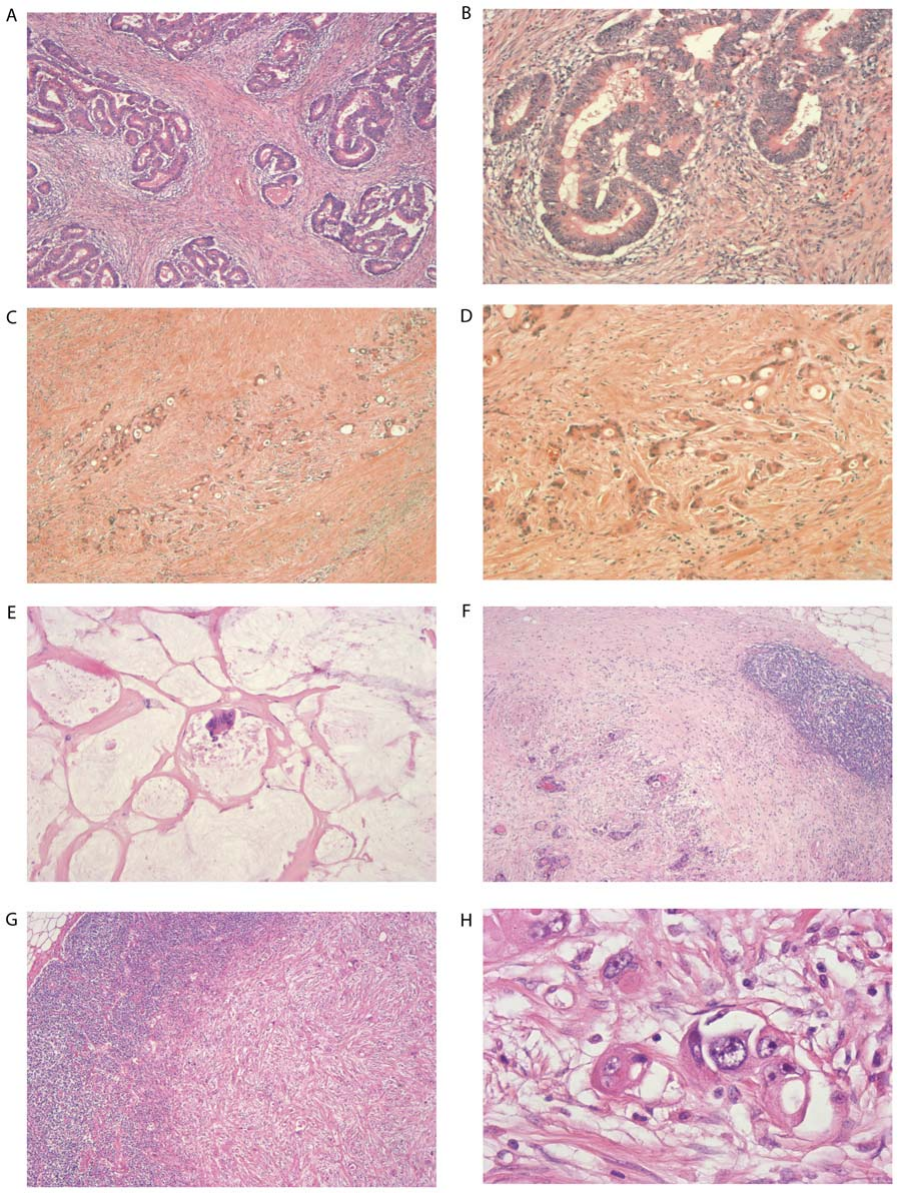
Stages of primary tumor regression and regional lymph node metastasis regression after neoadjuvant radiochemotherapy. A. HE stained rectal adenocarcinoma with tumor regression grade 1 (40x). B. HE stained rectal cancer tissue with tumor regression grade 1 (100x) C. Rectal carcinoma with tumor regression grade 2 (40x) D. Rectal adenocarcinoma with tumor regression grade 2 (100x). E. Tumor regression grade 3 (100x) in rectal cancer with extracellular mucin accumulation. F. Lymph node metastasis regression grade 2 (40x). G. Lymph node metastasis regression grade 3 (40x). H. Regional lymph node metastasis (400x) in standard HE staining.

**Figure 3:**
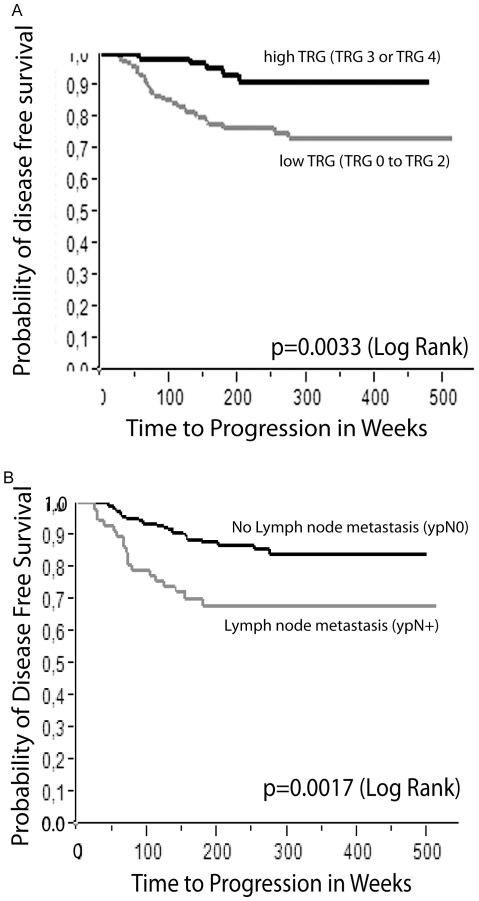
Kaplan Meier diagrams for the time to progression. A. Time to progression in weeks in relation to the probability of progression free survival stratified for a dichotomized tumor regression grading (TRG 0/1/2  =  no regression or dominant tumor mass with obvious fibrosis or mucin or dominantly fibrotic or mucinous changes, with tumor cells or groups; TRG 3/4  =  very few tumor cells in fibrotic or mucinous tissue or no tumor cells, only fibrotic or mucinous mass / total regression; according to Dworak et al. 1997). B. Time to progression in weeks in relation to the probability of progression free survival stratified for lymph node metastasis (ypN0 and ypN+).

A significant correlation was found between the grades of tumor regression (Fig. 2) and progression free survival when dichotomized for high and low tumor regression (p = 0.0033 Log-Rank) (Fig. 3A) in Kaplan Meier analysis. Multivariate analysis revealed a statistically significant difference when comparing TRG 1 and TRG 3 (Table 2). As in previously published reports [2], [3], [4], [5], [6], we also demonstrated that lymph node metastasis is an important independent prognostic factor in our cohort (p = 0.0017 Log-Rank) (Fig. 3B).

**Table 2:** Significant relationships as depicted in the multivariate Cox proportional hazard regression model.

Prognostic factors	Hazard Ratio	95% confidence interval	P-value
ypUICC stage I,II/III,IV	0.11	0.1828–0.7194	*0.0037*
Tumor regression TRG 1/TRG 3	3.31	1.2373–10.3846	*0.0163*
nodular LVD (nLV) absent (-)/present (+)	0.41	0.1896–0.8562	*0.0168*

### Association of peritumoral and intratumoral lymph vessel density with lymph node metastasis, UICC stage, and prognostic parameters

In a next step we evaluated the peritumoral and intratumoral lymphangiogenesis (pLVD and iLVD) and its relationship to metastasis in our cohort. Lymphangiogenesis was assessed using a commercially available antibody that detects podoplanin (D2-40, Dako) (Fig. 1B and 1C). The staining is specific for lymphatic endothelium and does not stain any blood vessels with detectable intraluminal erythrocytes. pLVD was not significantly correlated with lymph node metastases (p = 0.08 χ^2^). There was no significant correlation of pLVD and ypUICC stage or disease free survival. Additionally, iLVD did not correlate with prognosis, lymph node metastasis, or ypUICC stage.

### Association of intranodal lymphangiogensis in regional lymph nodes with UICC stage, progression free survival, and type of relapse

table-1-captionIn parallel, we assessed intranodal lymphangiogenesis by staining regional mesorectal lymph nodes with D2-40 as previously described. A lymph node is classified as positive for intranodal lymphangiogenesis when at least one lymph vessel can be detected in the follicular or/and interfollicular region of at least one mesorectal lymph node (designated as nLV+). In essence, in 102 patients all lymph nodes were negative for lymph node lymphangiogenesis, whereas in 43 patients in only one lymph node detectable lymphangiogenesis could be seen. In 58 patients more than one regional lymph node has detectable lymphangiogenesis.

**Figure 4:**
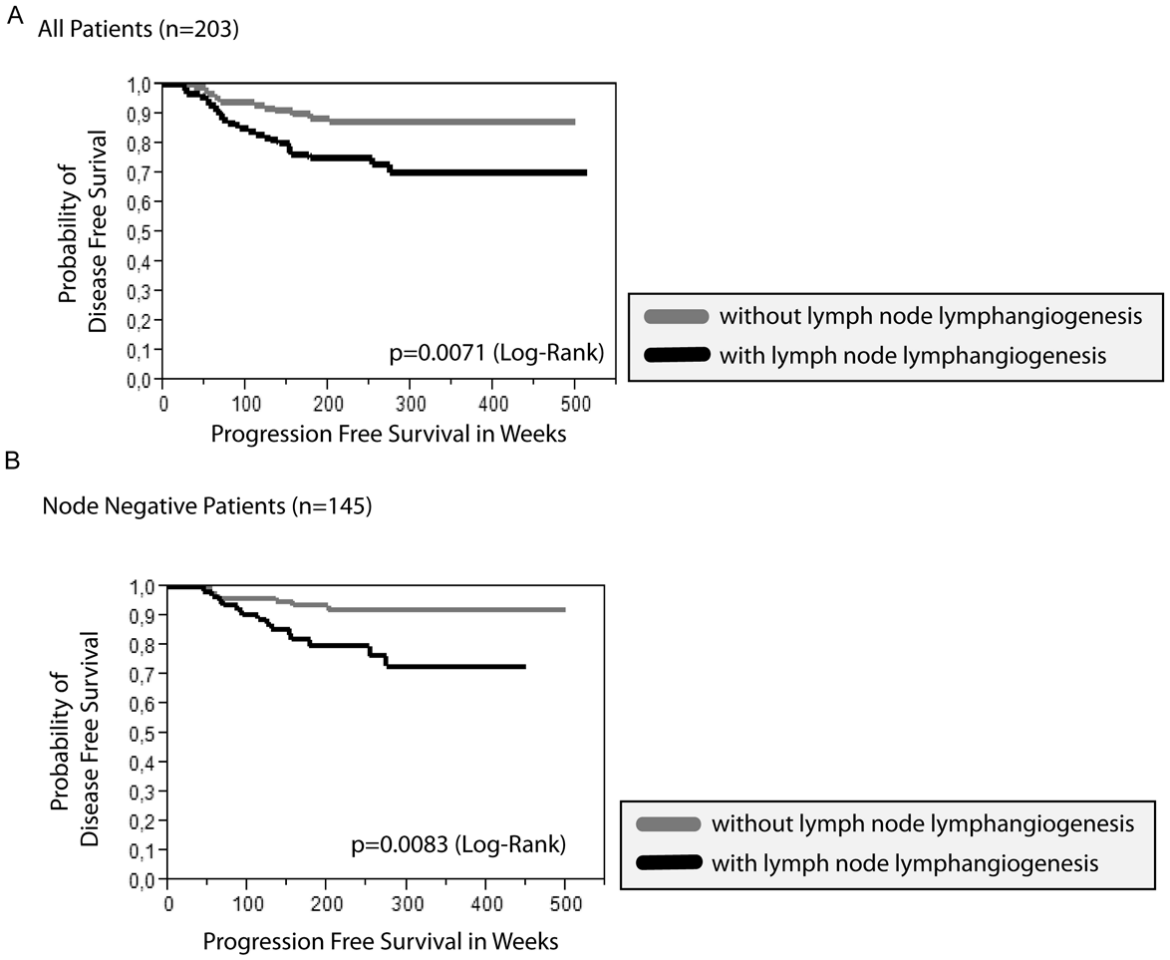
Lymph node lymphangiogenesis (nLV+) and its correlation with clinicopathological parameters and time to progression. “With lymph node lymphangiogenesis” is defined as at least one regional/mesorectal lymph node with detectable lymphangiogenesis in the follicular or interfollicular area. A. Time to progression in weeks in relation to the probability of disease free survival stratified for the presence of lymph node lymphangiogenesis in all patients. B. Time to progression in weeks in relation to the probability of disease free survival stratified for the presence of lymph node lymphangiogenesis in node negative patients.

**Figure 5:**
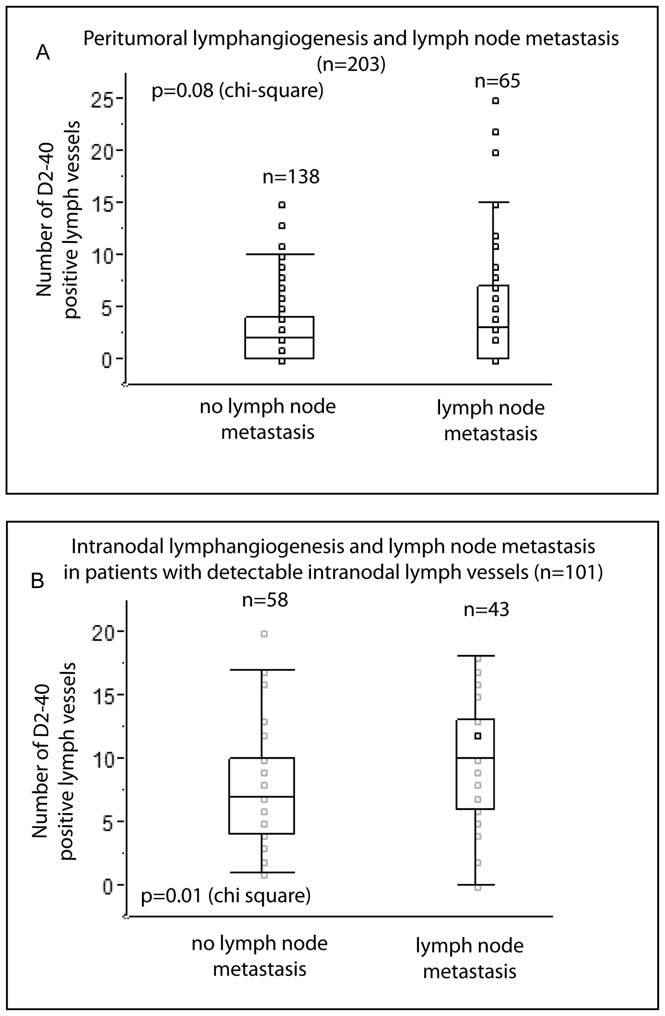
Lymphangiogenesis and metastasis. A. Box plots comparing the number of lymph vessels in the peritumoral region with the presence or absence of lymph node metastasis. B. Box plots comparing the number of regional lymph node lymph vessels in relation with the presence or absence of lymph node metastasis in patients with detectable lymph vessels in the regional lymph nodes.

We found a statistically significant correlation between nLV+ (lymphangiogenesis in at least one mesorectal/regional lymph node) and tumor relapse as well as ypUICC stage (Table 1). Kaplan Meier Analysis revealed a significant correlation of nLV+ with progression free survival time (p = 0.0071 LogRank) in the whole cohort (Fig. 4A). Furthermore, nLV+ was an independent prognostic factor as studied by the Cox proportional hazards regression model with the covariates ypUICC-stage, age, gender, tumor regression, intratumoral lymph vessel density, peritumoral lymph vessel density, and lymph node lymph vessel density (Table 2). Accordingly, subgroup analysis of node-negative patients presented a significant correlation between nLV+ and disease-free survival (Fig. 4B) using Kaplan-Meier analysis. When comparing the frequency of hepatic metastasis in patients with and without mesorectal intranodal lymphangiogenesis (nLV+), we found more hepatic metastasis in patients with nodal lymphangiogenesis (13 hepatic metastasis in patients with intranodal lymphangiogenesis versus 2 hepatic metastasis in patients without intranodal lymphangiogenesis). We also evaluated the number of lymph vessels (lymph vessel density, nLVD) detected in the regional lymph nodes. Here, the maximal number of lymph vessels detected in a lymph node with lymphangiogenesis correlates significantly with the node status (ypN0 or ypN+; p = 0.01 χ^2^) (Fig. 5B). In contrast, there was no correlation of the maximal number of lymph vessels with distant metastasis. When dichotomizing according to the median of the maximum lymph node lymph vessel density, we could not detect any significant correlation with time to progression. Also, the number of lymph nodes with lymphangiogenesis did not correlate with ypUICC stage or tumor relapse in the subgroup of patients with nLV+. In summary, the presence of regional lymph node lymph vessels represents an independent prognostic parameter, while the nodal lymph vessel density as well as the number of lymph nodes with detectable nodal lymphangiogenesis might be of minor importance for overall prognosis.

### Association of lymph node metastasis regression grading and prognosis

In our cohort, there was no statistically significant correlation between lymph node metastasis regression grading and tumor recurrence / disease free survival in the Kaplan Meier model in all patients and in subgroup analyses. In multivariate analyses no statistically relevant correlation could be found.

## Discussion

Regardless of tumor size and extent of invasion, regional lymph node metastasis (ypN+) is an important prognostic factor in patients treated with neoadjuvant radiochemotherapy [2], [3], [4], [5], [6]. Therefore, it is important to elucidate the mechanisms of lymph node metastasis as well as how lymph node metastasis might influence overall patient survival. Although controversial, peritumoral lymphatics are reported by some to play a role in promoting lymph node metastasis in colorectal carcinoma [10], [11], [12], [13] [12], [14], [15]. We used D2-40 antibody (Podoplanin, cell surface sialylated glycoprotein)[16], [17] to stain lymphatic vessels in primary adenocarcinomas and mesorectal lymph nodes of 203 rectal cancer patients. These patients were treated with preoperative radiochemotherapy followed by complete surgical removal of the tumor (R0). Our results indicate no significant correlation between peritumoral and intratumoral lymphangiogenesis and patient prognosis. Interestingly, we observed lymph node lymphangiogenesis was an important prognostic parameter in this patient cohort. As previously reported, lymphangiogenic growth factors such as VEGF-C and VEGF-A can migrate to the regional lymph nodes from primary tumors and promote lymphangiogenesis [7], [18]. We can predict a similar phenomenon in colorectal cancer as the level of VEGF-C was shown to be elevated in the blood serum of patients with colorectal cancer [19]. It has been hypothesized that lymph node lymph vessels can act as a metastatic niche for cancer cells [7], [8], [20], [21] and facilitate further metastatic transformation of the tumor cells. This might lead to therapy-resistant metastatic cancer and is therefore responsible for poor patient prognosis. Studies in other cancers also indicate a similar prognostic significance of lymph node lymphangiogenesis. In a B16 melanoma mouse model it was demonstrated that lymph node lymphangiogenesis is an early response to tumor growth and precedes tumor metastasis [22]. The significance of sentinel lymph node lymphangiogenesis in metastatic spread of breast cancer to non-sentinel lymph nodes has been shown in human patients. [23]. Similar results indicating the importance of lymph node lymphangiogenesis were reported in pancreatic cancer [8], squamous head and neck cancer [21], and extramammary Paget's disease [20].

Multimodal pretreatment of locally advanced rectal cancer has shown therapeutic benefits to the patients [24]. Previously, it has been shown that lymphangiogenic growth factors e.g. VEGF-C were upregulated by tumor cells during stress and promoted their stress-resistance ability [Bibr pone.0027402-Rinaldo1] [25] [Bibr pone.0027402-Rinaldo1] [26]. Therefore, it is possible that therapeutic stress during neoadjuvant treatment of rectal cancer enhances the level of synthesis of lymphangiogenic growth factors and increases the formation of lymph vessels in regional lymph nodes. This eventually leads to tumor recurrence and metastasis. Blocking the lymphangiogenic axis during neoadjuvant therapy should therefore improve the outcome of current treatment modalities for advanced stage rectal cancer. The association of lymph node lymphangiogenesis and worse prognosis further suggests the routine detection of lymph node lymphangiogenesis as an important parameter for the prediction of cancer relapse and as an indicator for a more aggressive adjuvant treatment.

In conclusion, our study demonstrates that the presence of intranodal lymphangiogenesis represents an independent prognostic marker associated with shorter progression free survival in patients with locally advanced rectal cancer, who are treated with neoadjuvant radiochemotherapy. Therefore, strategies to detect lymph node lymphangiogenesis in the clinical setting might help to identify patients who would benefit from more aggressive treatment options. Also, inclusion of a therapeutic approach to block lymphangiogenesis would be beneficial to neoadjuvantly treated advanced rectal cancer patients.

## Materials and Methods

### Patient material

Material used in this study is covered by the approval of ethic's committee of the University Hospital “Carl Gustav Carus”, TUD-Medical Faculty, for the use in research (EK59032007; “Feingewebliche und molekularpathologische Untersuchung in langzeit-archiviertem Gewebsmaterial des Instituts fuer Pathologie des Universitaetsklinikum Dresden/Medizinische Fakultaet Carl Gustav Carus, TU Dresden”; Chairman of the Committee: W. Kirch, MD). The decision of the ethic's committee specifically approved the use of tissue material for this minimally invasive and retrospective study that uses patient material that has been stored for more than three years. All patients consented to the use of tissue material for research (as noted in the files of the patients). According to the Ethic's committee oral consent is sufficient in this case (this consent procedure is approved by the Ethic's committee) because the material used here is not associated with the name of the patient and is coded. Two hundred and three patients (male: n  =  152, female: n  =  51, median age  =  70 years) with locally advanced rectal adenocarcinoma (UICC stage II/III) between 2001 and 2008 were included in this study (Figure 1). All patients underwent preoperative (neoadjuvant) 5-fluorouracil (5-FU) based radiochemotherapy and curative R0 surgery in analogy to the randomized phase III German Rectal Cancer Trials CAO/ARO/AIO-94 [1]. The data for disease relapses (distant metastasis or local recurrences) as well as other relevant data were retrieved from the database of the Institute of Pathology, University Hospital “Carl Gustav Carus”, Medical Faculty of the University of Dresden, Germany.

### Pathological examination of cancer specimen after neoadjuvant therapy

At least four samples were taken and evaluated from the primary rectal cancer. In the case of occult residual cancer during gross examination, the complete former cancer area was embedded and studied by histology. In case of the absence of residual cancer cells in the HE staining vital epithelial cancer cells were detected by cytokeratin staining. This procedure ensures that therapy induced tissue fibrosis as well as extracellular mucin accumulations were not considered as residual cancer tissue and incuded in the tumor regression grading. Mesorectal regional lymph nodes were dissected according to the “Union internationale contre cancer” (UICC). In case of examination of less than twelve regional mesorectal lymph nodes perirectal fatty tissue (minimum ten paraffin blocks) were embedded and evaluated for the presence of further lymph nodes that were not detectable by gross examination. To detect residual cancer cells Pancytokeratin staining was performed as described.

### Tumor and lymph node metastasis regression grading

Histopathological tumor regression (TRG) within the primary tumor was graded according to the semiquantitative system established by Dworak et al. [9]: TRG 0  =  no regression; TRG 1  =  dominant tumor mass with obvious fibrosis or mucin; TRG 2  =  dominantly fibrotic or mucinous changes, with few tumor cells or groups; TRG 3  =  very few tumor cells in fibrotic or mucinous tissue; TRG 4  =  no tumor cells, only fibrotic or mucinous mass / total regression [2] (Fig. 2).

Evidence of tumor response to preoperative radiochemotherapy within the perirectal lymph nodes was defined as the presence of nodal fibrosis or mucin pools (with or without residual tumor cells), as described previously [27], [28]. Lymph node metastasis regression (LRG) was evaluated with the same parameters like the primary tumor regression considering each metastatic lymph node: LRG 0  =  no regression; LRG 1  =  dominant tumor mass with obvious fibrosis or mucin; LRG 2  =  dominantly fibrotic or mucinous changes, with few tumor cells or groups; LRG 3  =  very few tumor cells in fibrotic or mucinous tissue; LRG 4  =  no tumor cells, only fibrotic or mucinous mass / total regression. Non-metastatic lymph nodes were distinguished from those with total metastatic regression (LRG 4) by absence of fibrosis or mucin (Fig. 2). When different LRGs were found in one patient, only the lymph node with the worst regression was considered.

### Immunohistochemistry

Immunohistochemistry was performed on 2 µm sections of paraffin-embedded blocks from the primary tumor and regional lymph nodes. Deparaffination, rehydration, and antigen retrieval were performed by steam cooking (98°C, 45 min) in PT Module buffer (pH 8.0) (Thermo scientific, Medac, Hamburg, Germany). The tissues were washed in Tris-buffered saline (TBS) (Thermo scientific, Medac, Hamburg, Germany) and incubated with monoclonal mouse anti human podoplanin detecting D2-40-antibody (DAKO, Hamburg, Germany, 1∶400 dilution) for 30 min at room temperature. Then, endogenous peroxidase activity was inhibited by incubating the slides in 3% H_2_O_2_ for 10 minutes. After washing in TBS, Ultravision LP large volume detection system HRP Polymer (Thermo scientific, Medac, Hamburg, Germany) was used according to the manufacturer's recommendation. The sections were stained with 0.5 mg/ml diaminobenzidin tetrahydrochlorid (DAB) and counter-stained with hematoxylin. In all experiments, positive and negative controls were included.

### Evaluation of immunohistochemistry

Lymph vessel density was assessed by the “hot-spot” method as described previously and was also referred to as the Chalkley point graticule method [29]. Briefly, all slides were scanned at low magnification (x40) and the areas with the highest density of D2-40 positive vessels were identified. Then, the number of D2-40 positive vessels was counted in those areas in five high-power fields (HPF, x400) by two independent pathologists (C.J. and M.H.M.) without knowledge of the clinicopathological findings, and the mean value of vessel counts were calculated. Intratumoral (lymph vessels within the tumor mass) and peritumoral (lymph vessels outside of the tumor, but within 2 mm from the tumor invasion front) LVD was determined separately. Vessels close to ulcerated areas were not counted. LVD was assessed on all dissected mesorectal lymph nodes including nodal metastases, residual lymphoid tissue of metastatic lymph nodes (if existing), and all non-metastatic lymph nodes. Preexisting intranodal vessels (intermediate as well as marginal sinus) were not counted. During evaluation high interobserver agreement was confirmed by using Cohen's Kappa coefficient (κ = 0.8; p<0.001). In case of interobserver differences, the case was reevaluated by simultaneous reassessment of the specimen.

### Statistical analysis

All tests are performed using SAS JMP Version 9. Associations between different categorical variables were assessed using the χ^2^ test. Continuous variables were compared using Mann-Whitney's U-test or ANOVA. Time to progression rates were compared using the univariate analysis of Kaplan-Meier, multivariate analysis was performed using the Cox proportional hazards regression model with the covariates ypUICC-stage, age, gender, tumor regression, intratumoral lymph vessel density, peritumoral lymph vessel density, lymph node lymph vessel density. P-values smaller than 0.05 were considered statistically significantly.
